# Supplementary material for: Evaluation of the Impact of the First Wave of COVID-19 and Associated Lockdown Restrictions on Persons with Disabilities in 14 States of India
Source: Int J Environ Res Public Health. 2022 Sep 9;19(18):11373. doi: 10.3390/ijerph191811373 (PMC9517051; doi:10.3390/ijerph191811373)
Supplement: Supplementary file 1 [file ijerph-19-11373-s001.zip › ijerph-1859168-supplementary.pdf]

**A STRATEGIC ANALYSIS OF IMPACT OF COVID-19 ON PERSONS WITH DISABILITY (PWD)**

**QUESTIONNAIRE (for the Person with Disability/Caregiver)**

**GENERAL:**

Name of the person interviewed:

Age: Sex:

Type of disability:

Occupation:

Date: Place:

Village: Taluka: District: State:

Name of the staff & CBM Partner:

-----------------------------------------------------------

1. What is your marital status?

   a) Never married b) Married c) Separated d) Divorced e) Widowed

2. Do you have children? a) Yes b) No

   If yes, How many?

   Participation

3. How much disability pension do you receive per month?

   Rupees ______________

   *Access*

4. Assistive Device:

   A) Do you use any assistive device? a) Yes b) No

   B) Which assistive device do you or PWD currently use?

   C) Do you/PWD sanitize it while using it? a) Yes b) No , if yes, How many times do you sanitize it in a day?

   D) How do you sanitize it (with what)?

**MEDICAL**

*Access*

5. Do you have any medical conditions?

a) Yes b) No

5.a. If yes_______________

6. Do you feel lockdown has made it difficult for you to get routine medical treatment?

a)Yes b) No

6.A Do you feel continuous lockdown will affect your health in future?
a) yes b) No

7. Did you face difficulty in accessing any of the following medical services during the lockdown?

A) Outpatient clinics – a) Yes b) No c) Did not need

B) Emergency medical services – a) Yes b) No c) Did not need

C) Medicines – a) Yes b) No c) Did not need

D) Physiotherapy / Paramedical services

a) Yes b) No c) Did not need

E) Regular blood pressure monitoring

a) Yes b) No c) Did not need

F) Regular sugar monitoring

a) Yes b) No c) Did not need

G) Surgical procedures

a) Yes b) No c) Did not need

8. Are you able to get the medicines you regularly take?

a) Yes b) No

9. Has lockdown affected your health insurance scheme?

a) Yes b) No c) do not have health insurance

10. Is online consultation helpful for you?

a) Yes b) No C) didn’t use it

11. Are you getting the same kind of care now, like before?

a) Yes b) No

*Access*

12. Have you postponed regular medical appointments because of lockdown?

a) Yes b) No

*<u>Participation</u>*

13. Have you had any medical condition where you had to postpone getting medical help because of lockdown?

a) Yes b) No

**REHABILITATION:**

*Participation*

14. Who helps you in your daily activities?

a) ASHA b) Paid caregiver c) Volunteer d) Family e) Others

f) do not require help

15. Which of the following therapies do you receive?

a) Physio b) OT c) Speech d) Prosthetics e) Others

f) do not require therapy

16. How do you receive therapy services during the lockdown?

a) In person at home b) In person at govt. rehab center

c) Private therapy center d) Virtual session e) Telephone session

f) Not receiving therapy due to lockdown

17. Do you think therapy /rehabilitation support services are available for PWD during the lock down?

a) Yes b) No

*Participation*

18. Do you think that you will be capable of managing yourself in case of another lockdown?

a) Yes  b) No  c) not sure

Access / Compassion / Network

19. Has the government given any special considerations to PWD during the lock down, especially in terms of access to vital information, rehab/therapy support services?

a) Yes  b) no

**MENTAL HEALTH**

20. What is bothering you the most since the lockdown

A. **Fear of Infection**
a) Not at all  d) Moderately  f) A lot

B. **Fear of infecting others**

a) Not at all  d) Moderately  f) A lot

**C. Fear of dying** a) Not at all  d) Moderately  f) A lot

D. **Lack of support** a) Not at all  d) Moderately  f) A lot

E. **Gender based violence** a) Not at all  d) Moderately  f) A lot

F. **Loss of income** a) Not at all  d) Moderately  f) A lot

G. **Interruption of care giver** a) Not at all  d) Moderately  f) A lot

**Any other** ____________________________

21. Since the COVID-19 outbreak, to what extent have you felt each of the following:

A) Stressed  a) Not at all  d) Moderately  f) A lot
B) Overwhelmed  a) Not at all  d) Moderately  f) A lot
C) Anxious  a) Not at all  d) Moderately  f) A lot
D) Uncertain  a) Not at all  d) Moderately  f) A lot

22. Have you experienced any of the following during the lockdown

a) Problems in relationship
b) Change in family dynamics
c) Abandonment

d) Isolation
e) Stigma
f) Violence
e) Discrimination
f) Others, Please specify ___________-

***Access***

23. Do you have access to information related to mental health & care, psychological or emotional support and services? Ex: Mental health helpline, online counselling, stress management tips etc.)

    a) Yes b) No c) do not need

24. Have you been able to get regular mental health counselling or therapy related services during the COVID-19 outbreak?

    a) Yes b) No c) Did not need

25. Did you face any problem for getting your regular psychiatric medicine if prescription date was old and you cannot get recent prescription?

    a) Yes b) No c) Did not need

Communication/Compassion/Participation

26. From whom are you getting emotional or practical support from family and friends during COVID-19 outbreak?

    Please specify ____________________

**Question -28 & 29 to be answered specifically by caregivers**

27. **As a caregiver**/parent, are you getting enough professional support like earlier during this covid19 outbreak?
    a) Yes b) No c) Did not need
    Participation/Compassion

28. **As a caregiver**, are you feeling stressed, anxious or depressed with caring for children or other family members at home with disability?

    a) Not at all d) Moderately f) A lot

29. **As a parent or caregiver**, did you feel unhappy when the therapy for your child had to be stopped during lockdown?
    a) Not at all d) Moderately f) A lot

_________________

## EDUCATION AND LIVELIHOOD

30. On being confined to the home, did the children feel distressed?

    a) Yes b) No

31. Since all schools are closed, has it affected the child learning?

    a) Yes b) No

*Access/Participation*

32. Is the school providing online teaching to children?

    a) Yes b) No

    32A. If yes, is it in accessible formats? Yes No

*Participation*

33. Has the lockdown impacted your daily activities pertain to livelihood?

    a) Yes b) No

Access/Participation

34. Has the lockdown affected the following

    A. Supply chain of material that you have developed?

    a) Yes b) No c) Cannot say

    B. supply of farm inputs like seeds, fertilizer and Pesticides?

    a) Yes b) No c) cannot say

    C. transportation of input and output supply due to lack of transportation?

    a) Yes b) No c) cannot say

    D. facility of water supply

    a) Yes b) No c) cannot say

35. Has the lockdown affected the Pensions / Remittance?

    a) Yes b) No c) Do not know

Participation/Network

36. Are you getting your full wages / pay during lockdown situation?

a) Yes b) No c) Not getting any wage

37. Are you borrowing money because of lockdown?

a) Yes b) No

For what : ____________________

*Communication*

38. Are you getting updates that you require for your business ( such as price, suppliers)

a) Yes b) No c) Not applicable

Access/Network

39. Do you think you will accept any job for low pay because of poor income after lockdown ends?

a) Yes b) No c) Not applicable

40. Are you able to sell your produce/products?

a) Yes b) No c) Not applicable

Networks/Access

41. Are loan/funds available with inclusive Cooperatives?

a) Yes b) No c) Not applicable

42. How are you ( person with disability) coping with the financial crisis situation ?

a) By reducing size and/or number of meals eaten in a day in order to have enough food to eat

b) By changing diet to cheaper or less-preferred foods to have enough food to eat

c) By selling off some household possessions and/or livestock in order to buy enough food to eat

d) By borrowing food or money for food from relatives, friends, or neighbors in order to have enough to eat

e) Not applicable

**SOCIAL EMPOWERMENT:**

<u>Network/Communication/Participation</u>

43. <u>Has lockdown affected your participation in the following</u>

A. Panchayat Raj Institution (PRIs) as Members?

a) Yes b) No c) Cannot answer

B. Village relief work?

a) Yes b) No c) Cannot answer

C. social mobilization with other community members for community issues?

a) Yes b) No c) Cannot answer

D. participation in village level covid response and planning?

a) Yes b) No c) Cannot answer

E. Disabled people organization (DPO) meetings?

a) Yes b) No c) Cannot answer

F. Do they feel that after lockdown, working with community and PRIs will change?

a) Yes b) No c) Cannot answer

**A STRATEGIC ANALYSIS OF IMPACT OF COVID-19 ON PERSONS WITH DISABILITY (PWD)**

**Interview Guide**

Name of the person interviewed:

PwD:

Age: Sex:

Designation/ Occupation:

Date: Place the person is from:

---

1. Please tell us about your disability, how are you managing it now during the COVID-19 outbreak and lockdown?
   Probe
   - What alternative arrangements have been made to meet these challenges?_ *Access*

2. Could you describe how the lockdown has impacted your daily life? Describe the biggest challenges you have faced during the lockdown. How are you dealing with this? *Compassion*
   Probe
   - How are you coping with the changes in your work and life due to COVID-19 outbreak?
   - Have you been able to buy essential commodities related to food, clean water supply, hygiene and other essential supplies?
   - Has the lockdown put extra burden on the family savings which was utilized for treatment?
   - How has life changed from pre-lockdown to lockdown?

3. For medical and chronic conditions for which you need medical care, how are you accessing medical services/treatment during this time? *Access*

4. If you need therapy on a routine basis, have you been able to get regular therapy related services during the COVID-19 outbreak?

5. Where do you get information related to symptoms of COVID-19? Is the information on COVID-19 in accessible format for you?
   What about information related to mental health care?
   Communication

6. How do you keep yourself away from COVID19 in terms of Self-Isolation, Social Distancing, Hand Hygiene wearing mask and Screening, especially given your disability?

7. Please tell us briefly about the psychological impact being isolated at home is having on you. For example, not being able to see friends or family and not being able to go out for exercise?

- What were the measures you took to reduce the stress or take care of your mental health?

8. Have you suffered any form of discrimination because of the COVID-19 outbreak? Have you suffered any form of violence because of the COVID-19 outbreak?

   Probe

   - Do you think being a women/man/girl/boy makes it easier or more difficult in the current context? Can you explain?

9. As a caregiver, are you experiencing any changes or challenges with caring for children or other family members with disability at home?

   Probe

   - Has it impacted the health of your child/maintaining hygiene measures to be taken up for your child and family?
   - As a parent or caregiver, how did you feel when the therapy for your child had to be stopped during lockdown?

10. Do you think the government has done enough for PwD during this time or could have done more, and do you have any suggestions to prevent negative experiences during future epidemics?

11. Are there any arrangements/specific plans/programmes been made to meet the needs of people with disabilities during this lock down by any organization/ institution? Did you receive this? Did you face any problem?

12. Do you think there are any positive impacts of the Covid and Lockdown?

**A STRATEGIC ANALYSIS OF IMPACT OF COVID-19 ON PERSONS WITH DISABILITY (PWD)**

**Interview Guide for Government officers and Project Managers**

Name of the person interviewed:

Government/ CBM Project Manager:

Age: Sex:

Designation/ Occupation:

Date: Place the person is from:

---

1. Do you think it is difficult for PWDs during COVID-19 outbreak and lockdown– how and why?
   Probe:
   - Can PwDs follow prevention techniques like Self-Isolation, Social Distancing, Hand Hygiene, wearing mask, etc
   - What about the psychological impact of being isolated at home is having on PwD. For example, not being able to see friends or family and not being able to go out for exercise

2. How do you expect PWD to manage their disability during the lock down, especially when services are not available and when they need it?

3. What do you think are the best ways to provide therapy/rehabilitation in case a PWD is confirmed positive of COVID-19 infection?

4. Do you think PWD will require special assistance in this kind of situation? What do you think will be there needs (Information, services-health rehab, general, Access, etc.)? Please explain.

   Probe
   - Do you have any suggestions for ensuring the needs of PWD are considered /addressed during situations like this?

5. What kind of arrangements/specific plans/programmes have been made to meet the needs of people with disabilities during this lock down in your department, organization or institution?

   Probe:
   - Has your department/organization done needs assessment during covid-19 outbreak?
   - Where do you get the information/ data/ records regarding PWD, and do you know of any other programs for PwD
   - Do you think you have sufficiently addressed the needs and concerns of PWD during the lock down with these arrangements/specific plans/programmes?

6. If there is another lockdown in the future, what kind of arrangements/specific plans/programs you think will help PWD in your locality?

7. What do you think are the best ways to include PWD in the actions to combat COVID19 and the Lock down? Please suggest

8. What steps should the government have taken to avoid hardship during lockdown?

9. Would you think the funds for PwD programs/ pensions would be impacted due to the pandemic, do you think the government will be able to allocate same amount of funds like last year?

10. Were there any issues related to abandonment, violence, abuse reported from beneficiaries during this lockdown?
